# Technical insights on adjustable velar extension and safety mechanism in Tübingen palatal plate for Pierre Robin sequence

**DOI:** 10.1016/j.jobcr.2024.12.021

**Published:** 2025-01-14

**Authors:** Hemwati Nandan, Bert Braumann, Teresa Kruse, Michael Wolf, Pragjyoti Jha, Srinivas Gosla Reddy, Prasad Nalabothu

**Affiliations:** a GSR Institute of Cranio-Maxillofacial and Facial Plastic Surgery, Hyderabad, Telangana, India; b Department of Orthodontics Centre of Dentistry University of Cologne, Germany; c Department of Orthodontics, Center for Rare Diseases University of Cologne, Germany; d Department of Orthodontics, University Hospital of RWTH Aachen, Germany; e Department of Paediatric Oral Health and Orthodontics, University Center for Dental Medicine UZB, Basel, Switzerland; f Department of Oral and Craniomaxillofacial Surgery, University Hospital Basel and University Children’s Hospital Basel, Basel, Switzerland

## Abstract

**Figure:**
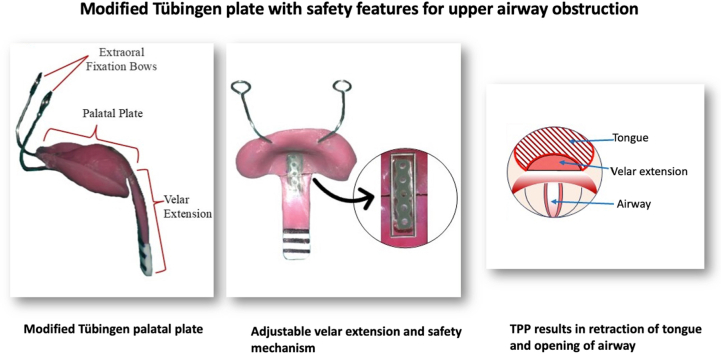

## 1 Introduction

Pierre Robin Sequence (PRS) is a congenital condition characterized by upper airway obstruction (UAO), accompanied by micrognathia and glossoptosis. Up to 90 % of cases also present with cleft palate, alongside feeding difficulties, especially in the neonatal period.^1^ PRS has a global birth prevalence of 9.5 per 100,000 live births.^2^ Management strategies range from prone positioning to surgical interventions like mandibular distraction osteogenesis, mandibular traction or tracheostomy.^3^

Mild cases are managed with prone positioning. However, this increases the risk of sudden infant death syndrome tenfold.^3^ The Tubingen Palatal Plate (TPP), a minimally invasive option, has demonstrated effectiveness in managing UAO for both isolated and syndromic PRS cases^4^^,^^5^. This technical note introduces a novel adjustable velar extension that customizes the TPP to patient-specific anatomy, enhancing safety and efficacy.

## 2 Materials and methods

### 2.1 Device design and modifications

The modified TPP builds on the original design introduced in 2003 at the Tubingen Craniofacial Center, Germany.^6^ It consists of the following components:

1. **Extraoral fixation Bows:** Stabilizes the plate by counteracting tongue pressure, preventing dislodgment (Fig. 1A)

**Fig. 1:**
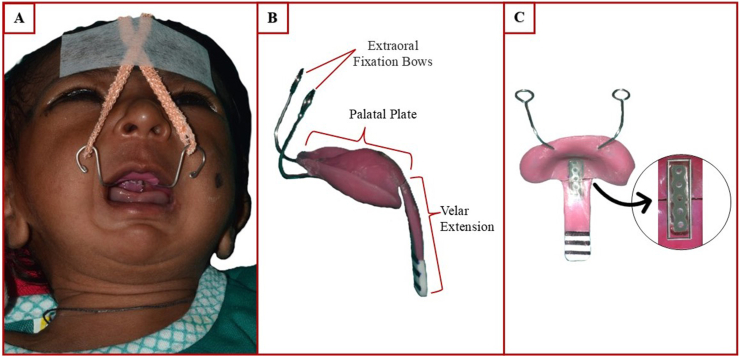
(A) A Robin sequence patient with inserted Tübingen Palatal Plate and extraoral adhesive tapes, (B) Tübingen Palatal Plate (C) Tübingen Palatal Plate reinforced with SS wire and elastomeric chain.
2. **Palatal Plate**: Similar to standard cleft palates, tailored to the infant’s anatomy (Fig. 1B).
3. **Velar extension**: A novel adjustable extension redirects the tongue forward, maintaining airway patency (Fig. 1B).
4. **Safety mechanism**: A stainless-steel wire and elastomeric chain secure the velar extension, preventing accidental dislodgment (Fig. 1C).

## 3 Technical insights

### 3.1 Adjustable velar extension and safety mechanism

The adjustable velar extension (baton) and safety mechanism comprise the following features:

• **Adjustable angulation**: A 0.036-inch stainless steel (SS) wire at the junction of plate and extension allows on-site adjustment of the angle to suit to individual needs. The velar extension is angled to reposition the tongue and widen the pharyngeal airway. If the angle is too shallow, it fails to open the airway. If too steep, it hinders swallowing. At any point during treatment, if we observe that the velar extension is not retracting the tongue forward adequately, the angulation is increased by carefully bending the stainless-steel wire
• **Safety mechanism**: An elastomeric chain is incorporated at the point of juncture between the palatal plate and the velar extension. The stainless steel wire enables the angle modification between the palatal plate and the pre epiglottic baton. Adequate space was created at the junction of the palatal plate and the velar extension to accommodate both components. Conversely, the acrylic of the plate and the baton is reinforced. In the event of the steel wire being severed, the elastomer chain holds both segments (palatal plate and velar extension) together (Fig. 1C). This mechanism prevents the baton from being aspirated or accidently swallowed, thereby protecting the infant from a life-threatening situation.

### 3.2 Angulation adjustment

The velar extension was angled to move the tongue forward and to widen the pharyngeal airway, thus it will relieve obstructive apnoea. The stainless-steel wire was positioned at the junction of the velar extension and the palatal plate, which allows for adjusting the angulation of velar extension at any time point during treatment, such as in the following situations.

A. In some cases, an infant may tolerate the TPP without any breathing difficulty but still be unable to feed properly, even after feeding training. A steeply angled velar extension may be the reason for this, as it restricts the necessary tongue movement during swallowing. In such cases, the angulation can be adjusted bedside, providing more space for the tongue to function properly.
B. When an infant is at ease with the TPP but encounters breathing difficulties during feeding. The possible cause could be an obtuse angle of the velar extension, which reduces the space between the velar extension and the pharyngeal walls, leading to respiratory distress during feeding. The angulation can be modified bedside or chairside whenever these symptoms are observed during treatment.

### 3.3 Length and width adjustment

**Fig. 2:**
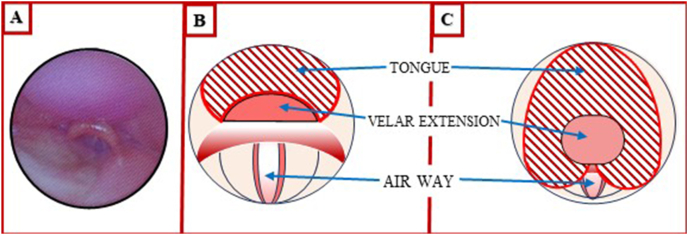
(A) Endoscopic view; velar extension positioned just superior to the epiglottis. (B) Optimal width of the extension retracts the tongue and open the airway. (C) Narrow velar extension fails to retract the tongue.

It is essential to conduct an endoscopic examination to assess the suitability of the Tübingen palatal plate. The dimensions of the palatal plate extension, specifically its length and width, are of paramount importance for ensuring optimal positioning and functionality. The velar baton terminates just above the epiglottis, thereby allowing for sufficient space for its movement. An excessively wide baton may induce discomfort and potentially cause soft tissue irritation of the lateral pharyngeal walls. In such cases, the palatal plate is not well-tolerated. Conversely, if the extension is too narrow, the tongue may move laterally, impeding the pharyngeal airway and preventing its full opening (Fig. 2). This can impair the efficacy of the Tübingen palatal plate.

## 4 Discussion

This technical note highlights the critical aspects of customizing the Tübingen Palatal Plate based on patient-specific anatomy for the treatment of newborns with PRS. Numerous studies have validated the TPP as a non-surgical intervention for UAO, demonstrating significant improvements in feeding and weight gain.^4^^,^^5^^,^^7^, ^8^, ^9^

Traditional TPP designs required a prototype to determine the optimal velar extension angle, necessitating additional working time and multiple try-ins. The incorporation of an adjustable stainless-steel wire streamlines this process, allowing direct adjustments without repeated fittings. While the appliance consists of two separate blocks connected by a stainless-steel wire, the elastomeric chain was included as a backup safety mechanism. In the unlikely event of wire breakage due to cold working, the elastomeric chain ensures that both segments remain connected, preventing any risk of detachment or protecting against life-threatening complications. This innovation aligns with contemporary trends in PRS management by prioritizing patient safety, procedural efficiency, and treatment efficacy.

## 5 Conclusion

The novel adjustable velar extension with an integrated safety mechanism in the TPP represents a significant advancement in PRS treatment. This modification eliminates the need for prototyping, reduces laboratory burden, and enhances appliance safety and functionality. Its ease of use and cost-effectiveness make it a promising addition to the clinician’s toolkit.

## Declaration of competing interest

The authors declare that they have no known competing financial interests or personal relationships that could have appeared to influence the work reported in this paper.
